# Layered Cuprates Containing Flat Fragments: High-Pressure Synthesis, Crystal Structures and Superconducting Properties †

**DOI:** 10.3390/molecules26071862

**Published:** 2021-03-25

**Authors:** Evgeny M. Kopnin

**Affiliations:** Pirelli Tyre SpA, Via Piero e Alberto Pirelli, 25, I-20126 Milan, Italy; evgeny.kopnin@pirelli.com

**Keywords:** cuprates, layered structures, high-pressure, superconductivity, homologous series

## Abstract

High-pressure synthesis and crystal structures of the homologous series AuBa_2_(Ca,Ln)_n−1_Cu_n_O_2n+3_ (*n* = 1–4; Ln = rare-earth cations) are described. Their crystal structures and superconducting properties are compared with the corresponding members of the Hg-homologous series. Numerous cuprates containing flat structural fragments (CuO_4_, CO_3_ and BO_3_) synthesized mainly at high pressure are compared in terms of structural peculiarities and superconducting properties. Importance and future prospects of high-pressure application for the preparation of new superconducting oxides are discussed.

## 1. Introduction

Numerous high-temperature superconducting cuprates have complex perovskite-based intergrowth crystal structures. In these structures, it is possible to select different blocks, for example, perovskite-like (CaTiO_3_), halite-like (NaCl), fluorite-like (CaF_2_) and/or other ones stacking along the four-fold axis of the perovskite sublattice [1]. The first member of series of Ruddlesden-Popper (RP) phases La_2_CuO_4_ has a simple intergrowth structure consisting of perovskite-like and halite-like fragments (K_2_NiF_4_ structural type). High-temperature superconductivity at T_c_ = 36 K was discovered in this material after hole doping by Ba^2+^ [2]. T_c_ was shown to increase up to 52.5 K when measured in situ under a hydrostatic pressure of 1.68 GPa [3].

**Figure 1:**
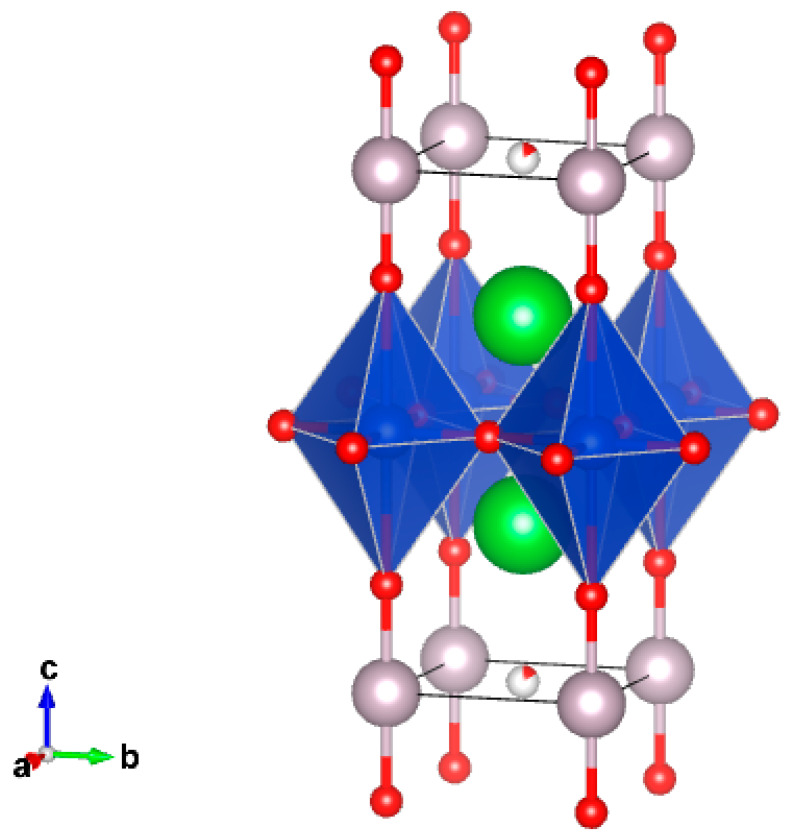
Crystal structure of Hg-1201. The coordination polyhedra of copper (distorted octahedron) and Hg (dumbbell) are shown.

The crystal structures of Hg-containing series of superconducting cuprates, HgBa_2_Ca_n−1_Cu_n_O_2n+2+δ_ (*n* = 1–6), can be described as intergrowth ones [4,5,6,7,8,9]. Defect halite-like fragments and perovskite-like fragments are stacking along the four-fold axis, which coincides with the crystallographic *c*-axis (Figure 1). Hg^2+^ cations have typical dumbbell-like coordination with a significant range of oxygen stoichiometries in the HgO_δ_ layers. In the crystal structures of compounds with *n* = 2–6, the corresponding amounts of CuO_2_ layers are separated by Ca^2+^ cations forming repeated perovskite-like blocks. It is important to note that the highest T_c_ (up to 138 K) was reported for the compound with *n* = 3. Moreover, at ultrahigh pressures above 20 GPa T_c_ increased remarkably and reached 166 K in fluorinated Hg-1223 [10,11,12].

Since the first high-pressure device was invented by Bridgeman in the 1940s, high-pressure has been extensively used in solid state sciences. It is established that ultrahigh pressure deals with the pressures greater than 1.01 GPa, and now the pressure scale is extended up to 500 GPa at temperatures exceeding 3000 °C. The most frequently used apparatus in high pressure research is the diamond-anvil cell (DAC) in a piston-cylinder design. It allows one to achieve pressures as high as 4 GPa or even more. The disadvantage of the Bridgeman device in a piston-cylinder design is the small sample size. That problem was solved by the belt-like apparatus invented in the 1960s, that can generate 10 GPa at temperatures up to 2000 °C. The modern devices are multianvil cells, where the anvils are usually made from materials with high hardness, preferably from diamond. Detailed descriptions and a comparison of different high-pressure apparatus are given in a recent review [13].

This review is dedicated mainly to the application of high pressure for the synthesis and superconducting properties optimization of layered cuprates. However, superconductivity at T_c_ > 200 K in H_3_S (T_c_ = 203 K at 150 GPa) [14] and in LaH_10_ (T_c_ ~ 250 K at 170 GPa) [15] was recently discovered. These new exciting materials belong to conventional superconductors with properties that can be described by the Bardeen–Cooper–Schrieffer and the Migdal–Eliashberg theories. In addition, first-principles calculations based on density functional theory and recently developed non-adiabatic theory are used for explanation and prediction of properties of new superconducting materials [16,17,18,19,20,21,22,23]. The latest results increase significantly interest in high pressure research and one can hope for further rapid progress in this direction.

## 2. Au-Containing Cuprates: High-Pressure Synthesis and Characterization

**Figure 2:**
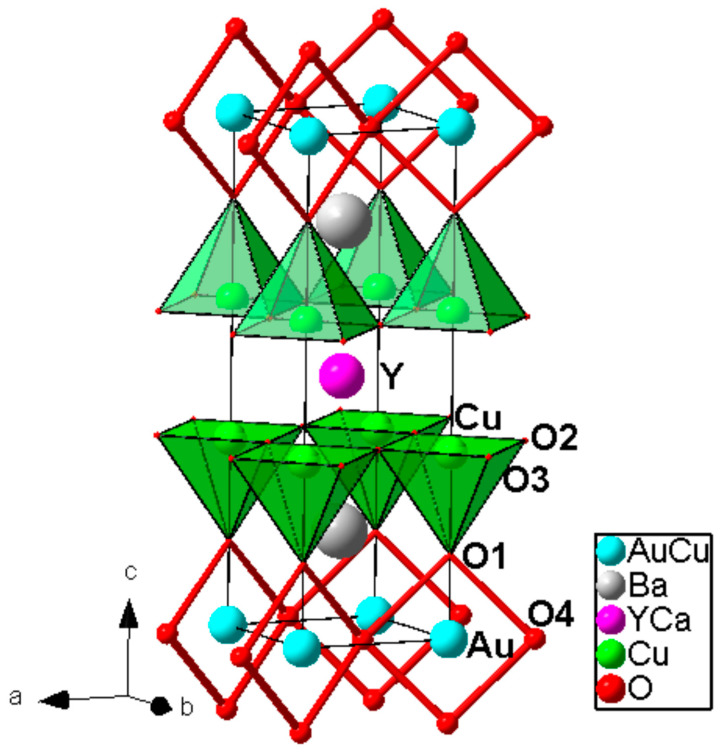
Crystal structure of the Au-1212 compound. Zigzag chain of AuO4 square-planar units is selected. Reproduced with permission from [9].

High pressure applications is strongly required for the synthesis of these materials because it is only way to stabilize gold oxides which are thermally very unstable at ambient pressures. The general formula of the homologous series is as follows: AuBa_2_(Ca,Ln)_n−1_Cu_n_O_2n+3_ (*n* = 1–4; Ln = rare-earth trivalent cations). The second member AuBa_2_Y_1−x_Ca_x_Cu_2_O_7_ was synthesized in polycrystalline and single crystal form at 1.8 GPa/950 °C and at 6 GPa/1380 °C, respectively [24,25]. Its crystal structure was determined from single crystal X-ray diffraction data in space group Pmmm (Jana 2000 software; R, R_w_ (I >3 σ (I)) = 0.0260, 0.0219; R, R_w_ (all data) = 0.0443, 0.0225; the details are given in [25]); a = 3.8260(2) Å, b = 3.8501(2) Å, c = 12.075 (1) Å. The samples exhibited bulk superconductivity with T_c_ = 82–84 K. The crystal structure of Au-1212 compound is shown in Figure 2.

The cation stoichiometry Au: Ba: Y: Ca: Cu = 0.8(1): 1.9(1): 0.7(1): 0.4(1): 2.2(1) obtained from electron probe microanalysis (EPMA) data is in agreement with that obtained from X-ray single crystal diffraction, indicating ca. 20% substitution of Cu^1+^ cations on the Au^3+^ site, it was associated with approximately the same amount of oxygen vacancies in the corresponding plane. It should be noted that no superstructure related to possible Cu/Au ordering was revealed from the X-ray single crystal diffraction data.

Au^3+^ is located in square-planar coordination with Au-O distances 1.980 Å and 2.027 Å. AuO_4_ squares share corners and they are forming zigzag chains along the short *a*-axis, and that is different from YBa_2_Cu_3_O_7_ where CuO_4_ squares ran along the longer *b*-axis. The coordination of the in-plane Cu is square pyramidal with an apical distance of 2.41 Å, it is typical for layered cuprate superconductors.

The third and fourth members of the Au-series, Au_1+x_Ba_2_Ca_2_Cu_3−x_O_9_ and AuBa_2_Ca_3_Cu_4_O_11_, were synthesized at 6 GPa and 1250–1300 °C [26]. They crystallize in primitive orthorhombic unit cells with a = 3.8182(4) Å, b = 3.8555(4) Å, c = 15.445(2) Å and a = 3.8266(3) Å, b = 3.8505(3) Å, c = 18.494(1) Å, for Au-1223 and Au-1234, respectively.

**Figure 3:**
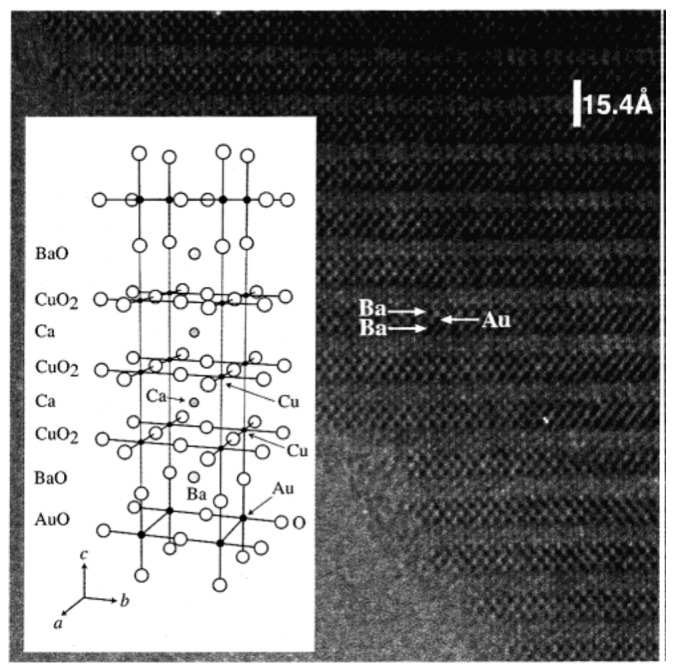
HRTEM image of Au-1223 phase taken along (100) zone axis. The structure model of Au-1223 is shown in the inset. Reproduced with permission from [10].

A HRTEM image of Au-1223 phase along the *b*-axis is shown in Figure 3. It is confirmed the 1223 stacking along the *c*-axis: BaO-AuO-BaO-CuO_2_-Ca-CuO_2_-Ca-CuO_2_. Attempts to synthesize next members (*n* = 5, 6) were not successful both at 1.8 GPa and at 6 GPa. AuBa_2_Ca_3_Cu_4_O_11_ showed bulk superconductivity with T_c_ ~ 99 K, but Au-1223 showed only a weak diamagnetic signal at T_c_ ~30 K. In order to understand the possible reason, crystal structure of Au-1223 was determined from single crystal X-ray diffraction data (SHELXL-97 software; S.G. Pmmm; R1, wR2 (I > 2 σ (I)) = 0.0188, 0.0437; R, R_w_ (all data) = 0.0301, 0.0484; the details are available in [27].

The cation stoichiometry Au: Ba: Ca: Cu = 0.9(1): 1.91(6): 2.10(6): 3.1(1) was obtained from EPMA. The crystal structure is similar to Hg-1223, but the cation stoichiometry is indicative of some possibility of Cu/Au disorder. The refinement of the occupation yielded 4.1% of Cu in Au site. Au-O in-plane distance is 2.055 Å and it is noticeably longer than typical Cu-O in-plane distances that correlate with their ionic radii difference.

Contrary to Hg-1223, oxygen in the AuO layer is located at (x,1/2,0), it leads to formation of AuO_4_ zigzag chains as in Au-1212. Electron diffraction revealed the weak superstructure with *b_s_* = 2b in Au-1223, however, the corresponding intensities in X-ray single crystal diffraction data were extremely weak. This suggests no ordering of zigzag-like chains and/or their irregularity.

Related to the suppression of superconductivity, some incorporation of Au for Cu in the CuO_2_ planes (6–8%) should be taken into consideration. Although the degree of the substitution is very moderate, even this level can lead to superconductivity suppression.

**Figure 4:**
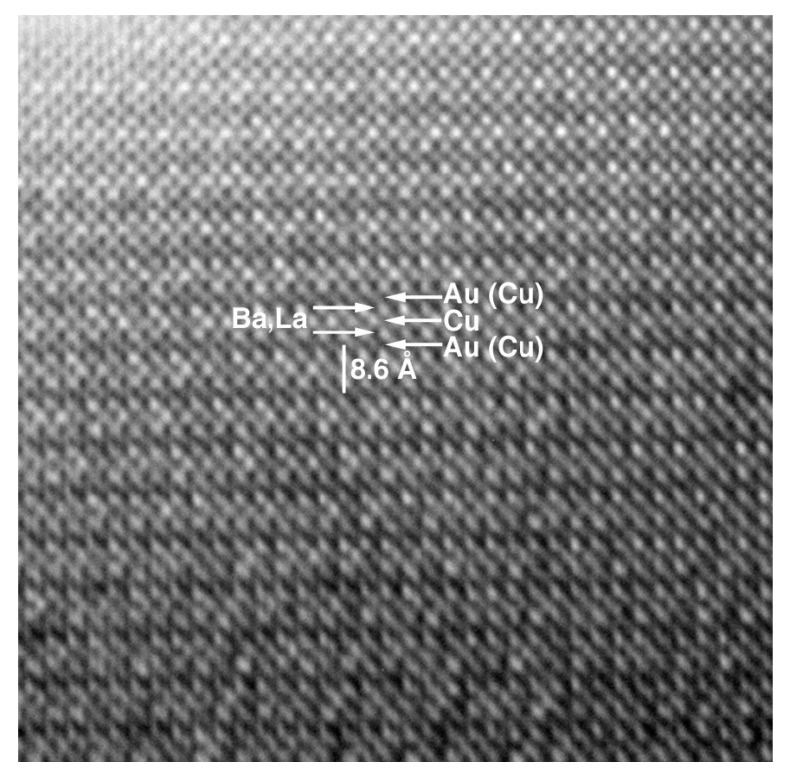
HRTEM image of Au-1201 compounds taken along (010) zone axis. Reproduced with permission from [12].

Au-1201 is the less known member of the Au-series. Au(Ba,La)_2_CuO_5±δ_ was obtained as a principal phase in the polyphasic sample prepared at 6 GPa and 1250 °C [28]. It crystallizes in an orthorhombic primitive cell with the parameters a = 3.7976(3) Å, b = 3.8509(3) Å, c = 8.5749(9) Å. EPMA shown the cation stoichiometry Au: Ba: La: Cu = 0.75(8): 0.8(1): 1.2(1): 1.25(8), therefore, the approximate formula of Au-1201 can be expressed as Au_0.75_Ba_0.8_La_1.2_Cu_1.25_O_5±δ_. Therefore, it suggests that ca. 25% of Au positions are occupied by Cu. HRTEM indicated that this is a first member of AuBa_2_(Ca,Ln)_n−1_Cu_n_O_2n+3_ series (Figure 4). However, an ED study revealed a weak superstructure with *a_s_* = 5a, *b_s_* = b, *c_s_* = 2c. It could be due to possible Au/Cu ordering. Samples of Au-1201 showed a weak diamagnetic signal with T_c_ = 19 K and low level of diamagnetic Meissner fraction.

## 3. Au and Hg-Containing Series: Structural Details and Superconductivity

Crystal structures of corresponding members of Au and Hg-series are rather similar (except for the first member), however, there are some important differences. First, maximum T_c_ was achieved in the Hg-series but not in the Au-series [26,29]. Moreover, only first four members of Au-series were identified in comparison with six members of Hg-series (the *n* = 7 member was obtained only if doped in the Hg site). The possible reason could be a noticeable mismatch between Au-O and Cu-O in-plane distances that lead to formation of zigzag chains consisting of corner-shared AuO_4_ square-planar units along *a* and/or *b* crystallographic axis. That mismatch can impede the formation of higher members of this series and their synthesis may require higher pressures application.

The extra oxygen in HgO_δ_ layer of Hg-series members is located in the central position (1/2,1/2,0) and is very weakly bonded with Hg. This results in a rather extended oxygen stoichiometry range and a possibility to optimize T_c_ using various thermal treatments of these materials [4,5,6,7]. Oxygen in Au-containing layers is located in more general positions and the oxygen stoichiometry in Au-series members is practically fixed and can vary in the very limited range only due to some doping caused by Cu incorporation resulting in Au/Cu disorder in Au-O plane. It may create serious difficulties to achieve the appropriate hole concentration, and, therefore, for T_c_ optimization.

It should be particularly taken into account some extent of Au incorporation in CuO_2_ layers that was not revealed for Hg in Hg-series. It is a possible reason of suppression of bulk superconductivity in Au-1201 and Au-1223. In Au-1201 it might be also due to an insufficient hole concentration. Additional studies preferably based on X-ray single crystal diffraction data are required to verify these hypotheses.

The ultimate member (*n* = ∞) of both series, CaCuO_2_, has so-called “infinite-layer” structure, which is very simple. It contains only corner-shared square planar CuO_2_ units and its unit cell consists of only two stacking layers (Ca^2+^) (CuO_2_) along the *c*-axis. At ambient pressures, this structure is stable in the very narrow x range of Ca_1-x_Sr_x_CuO_2_ solid solution (0.10 ≤ x ≤ 0.16) [30]. High-pressure application results in extension its stability range up to 0 ≤ x ≤ 1. Syntheses of CaCuO_2_ with “infinite-layer” structure were reported by using a belt-type apparatus at 6 GPa and high Ar pressure of 1,4–1,6 GPa [31,32]. Using the last technique, single crystals were grown. It showed a weak diamagnetic signal at T_c_ = 78 K, however, superconducting transition was not supported by the resistivity measurements and was attributed to some A-deficient domains. Bulk superconductivity was observed only after the electron doping by large rare-earth cations.

It should be noted that the “infinite-layer” structure was also reported for reduced nickelates as LaNiO_2_ [33] and Nd_1−x_Sr_x_NiO_2_ [34] containing Jahn-Teller cation Ni^1+^. These compounds were prepared using soft chemistry methods and they did not show superconducting properties.

## 4. La_2_CuO_4_ -Related Superconductors in Sr-Cu-O and Ba-Cu-O Systems

Sr_2_CuO_3_ crystal structure is stable at normal pressure and is heavily oxygen deficient. Cu is in square-planar coordination and CuO_2_ units are corner-shared along the *a-*axis [35]. At pressures as high as 6 GPa, 800–900 °C and in a strongly oxidizing atmosphere (created by adding KClO_4_) it was reported to transform into a layered structure of K_2_NiF_4_ structural type related to La_2_CuO_4_ and, moreover, a new series of copper oxide superconductors Sr_n+1_Cu_n_O_2n+1+δ_ was discovered [36]. The initial stoichiometry of the *n* = 1 member corresponds to Sr_2_CuO_3.1_, and the main peaks were indexed in tetragonal unit cell with a = 3.764 Å, c = 12.548 Å. A superlattice with *a_s_* = 4 × √2a, *c_s_* = c was revealed from electron diffraction data. The member with n = 2 was characterized but it was not obtained as single or dominant phase sample. According to X-ray diffraction data, it has a tetragonal unit cell with a = 3.902 Å, c = 21.085 Å. The samples exhibited bulk superconductivity at 70 K and a weak diamagnetic signal at ~ 100 K was attributed to a minor phase, a series member with *n* = 2. Higher members (*n* = 4, 5) were identified by electron diffraction but were not reported in bulk form.

Another research group published the synthesis of Sr_2_CuO_3+δ_ at 5.7 GPa and 900 °C using KClO_4_ as an internal oxidizer [37]. The as-prepared material was nearly single phase with unit cell parameters a = 3.7556(3) Å, c = 12.521(2) Å (*I4/mmm*) and it exhibited bulk superconductivity at T_c_ = 70 K. No X-ray evidence of other series Sr_n+1_Cu_n_O_2n+1+δ_ members was found. Heat treatment under flowing N_2_ and 310 °C for 1 h resulted in enhancement of T_c_ up to 94 K, unit cell parameters of Sr_2_CuO_3+δ_ changed to a = 3.7591(1) Å, c = 12.518(9) Å. Based on these data, the as-prepared sample is considered to be in an overdoped state, so δ > δ_opt_ [38].

The average crystal structures of superconducting and non-superconducting samples of Sr_2_CuO_3+δ_ with the tetragonal unit cell were refined using neutron powder diffraction data [39]. Superconducting sample (T_c_ = 65 K, Meissner fraction volume ~ 6%, δ ~ 0.1) was prepared by treatment of orthorhombic Sr_2_CuO_3_ at 6 GPa and 850 °C using an internal oxidizer KClO_3_. Tetragonal normal pressure phase Sr_2_CuO_3+δ_ was synthesized in flowing oxygen at 350 °C from hydroxometallate precursor Sr_2_Cu(OH)_6_, the obtained material did not show any diamagnetic signal above 4 K. In both crystal structures oxygen vacancies in CuO_2_ layers were revealed. It can be a reason why both samples did not exhibit bulk superconductivity. A weak diamagnetic signal in the “high-pressure” sample can be attributed to some superconducting clusters without these vacancies. However, more experimental data are required for verification of this assumption.

Another explanation of superconductivity in Sr_2_CuO_3+δ_ (δ ~ 0.1–0.6) was proposed in later publications [40,41]. Sr_2_CuO_3+δ_ single phase samples were prepared at 6 GPa and 1100 °C. Only La_2_CuO_4_-like phase was detected. As a source of oxygen SrO_2_ was used instead of KClO_4_ or KClO_3_, as it permits one to avoid the formation of another superconducting phase (Sr,K)CuO_2_Cl_2_ as an impurity [42]. Then the samples were annealed at different temperatures under flowing N_2_. The best superconducting properties were achieved for the samples with nominal *δ* = 0.4 that exhibited the highest T_c_ = 75 K.

The TEM and electron energy loss spectroscopy (EELS) techniques have been utilized to characterize superconducting Sr_2_CuO_3+*δ*_ (with nominal *δ* = 0.4) samples, both in the as-prepared state and post-annealed at different temperatures in order to reveal the superconducting phases. It was demonstrated that with raising the annealing temperature, T_c_ increased from 75 K (as-prepared) to 89 K (post-annealed at 150 °C) and 95 K (post-annealed at 250 °C). In the last stage the superconductivity disappeared when the annealing temperature exceeded 250 °C. TEM investigations revealed two types of modulated phase, *Fmmm* and *C*2/*m*, in the sample. The structure phase transitions are as follows. It starting from *C*2/*m* modulated phase → *Cmmm* modulated phase (annealed at 150 ◦C) → *Pmmm* modulated phase (annealed at 250 ◦C) → unmodulated orthorhombic structure (annealed at 350 ◦C), while no obvious changes are found for the *Fmmm* modulated phase up to 250 ◦C. The experimental results show that the *C*2/*m* modulated phase is responsible for T_c_ at 75 K, the *Cmmm* modulated phase exhibited T_c_ at 89 K, and for the *Pmmm* modulated phase T_c_ was observed at 95 K. *Fmmm* modulated phase is non-superconducting. That was attributed to the oxygen vacancies located at the Cu-O planes. It was concluded that, besides the hole doping level, the reordering of apical oxygen in those superconducting modulated phases is the important factor that governs the T_c_ in Sr_2_CuO_3+δ_. Moreover, Ba-substitution led to increase T_c_ up to 98 K in Sr_1.4_Ba_0.6_CuO_3+δ_ [43].

The detailed analysis of structure and superconducting properties in Sr_1−x_Ba_x_CuO_3+δ_ system was performed in [44]. The authors pointed out that there is no direct experimental evidence of the absence of oxygen vacancies in Cu-O planes. On the contrary, there is a clear evidence of oxygen vacancies (estimated in ~30%) from neutron powder diffraction data [39]. The simulation of the electron diffraction data also revealed an average commensurate superstructure (*Fmmm*) with orthorhombic supercell 5√2*a*_p_ × 5√2*a*_p_ × *c*_p_ (p belongs to tetragonal *I4/mmm* K_2_NiF_4_-type structure) and two different Cu-Cu distances in Cu-O plane [45,46]. This is in agreement with the oxygen vacancies in this plane deduced from with the neutron diffraction data. It is remarkable that T_c_ is almost triple in comparison with La_2_CuO_4_-based superconductors [2,47], therefore, the author concluded the existence of an enhancement of superconductivity.

Two new possible mechanisms of enhanced superconductivity were assumed: negative U-centers and optimum inhomogeneity. The concepts of negative U-centers deal with a band of paired electrons formed by the overlap of negative −U oxygen vacancies [48,49,50]. Optimum inhomogeneity considers pairing in the CuO_2_ layer that may be enhanced by an optimal inhomogeneity distribution of oxygen sites and/or by a clustering of vacancies rich and vacancies poor regions [51,52].

Recently, bulk superconductivity with T_c_ > 70 K was discovered in a new layered cuprate Ba_2_CuO_4−y_ (nominal content y ~ 0.8) [53,54,55]. It is synthesized at 18 GPa and 1000 °C in a highly oxidizing atmosphere. It should be noted that this material is very hygroscopic and cannot be prepared at lower pressures. Surprisingly, the exhibited T_c_ is more than 30 K higher than for doped La_2_CuO_4_ and superconducting volume fraction reaches as high as 30%. This evidence for bulk superconductivity was also confirmed by the muon spin rotation (µSR) showing approximately 40% superfluid density and the specific heat measurements. X-ray diffraction showed La_2−x_Sr_x_CuO_4_-like structure (I4/mmm) and the Rietveld refinement yields the lattice parameters of the compound with *a* = 4.003 Å and *c* = 12.94 Å at room temperature, respectively.

Moreover, it was demonstrated that this new cuprate has some peculiar features making it different from “conventional” cuprate superconductors: (a) the apical oxygen distance is significantly shorter than that known for all other cuprates so far; (b) superconductivity occurs at very high hole doping. It should be considered as strongly overdoped state in comparison to the optimal value of p~0.14–0.15 for the previously known “conventional” high T_c_ cuprates; (c) its structure contains numerous oxygen vacancies, presumably located in Cu-O plane. Moreover, Cu-O in-plane distances are 2.00 Å and longer than apical ones (1.86 Å), i.e., the defect octahedron is very compressed [56,57,58]. It is shown by X-ray absorption measurement that in the compressed octahedron, the 3dz^2^-r^2^ orbital should be lifted above the 3dx^2^-y^2^ orbital resulting in significant 3D nature in addition to the conventional 3dx^2^-y^2^ orbital [59]. Based on these data, it was suggested that Ba_2_CuO_4-y_ is a member of a different branch of high-T_c_ cuprate materials [60,61]. Another example of cuprate synthesized at high-pressure is Cu_0.75_Mo_0.25_Sr_2_YCu_2_O_7.54_, it has crystal structure similar to YBCO123 phase and exhibited T_c_ at ~ 87 K being strongly overdoped (nominal content p ~ 0.46) [62,63].

It can be concluded that further increase of high pressure may result in the discovery not only of higher members of known superconducting series, but also some new superconducting materials with high T_c_ and possibly new original superconductivity mechanisms.

## 5. Cuprates with Edge-Shared CuO_4_ Units in Sr-Cu-O and Ca-Cu-O Systems: Spin-Ladder and Infinite-Chain Composite Structures

**Figure 5:**
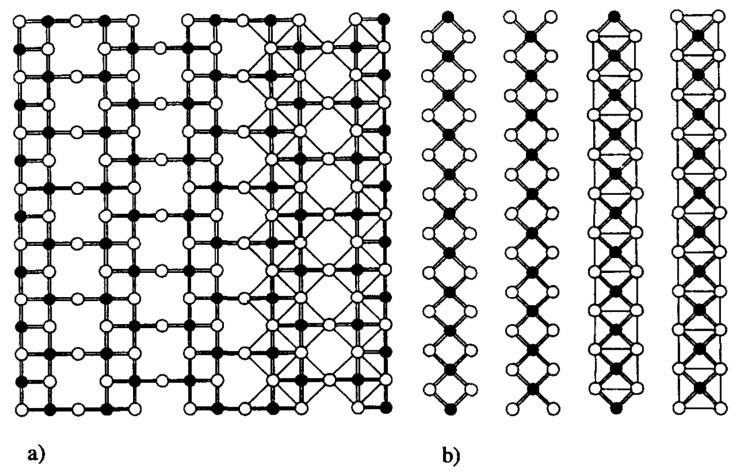
Two different Cu-O planes in the structure of Sr_14_Cu_24_O_41_: (**a**) two-leg ladder, (**b**) simple edge-sharing CuO_2_ chains. Reproduced with permission from [70], Figure 1.

Numerous cuprates contain edge-shared (CuO_2_) square-planar units. An interesting example is the series of so-called spin-ladder cuprates. They belong to the homologous series Sr_n−1_Cu_n+1_O_2n_ (n = 1, 2) [64]. These compounds can only be synthesized under high pressures. It should be underlined that at normal pressure the stable compounds in SrO-CuO system are SrCuO_2_ [35], Sr_2_CuO_3_ [65] and Sr_14_Cu_24_O_41_ [66,67]. SrCuO_2_ crystal structure is quasi-one-dimensional (1D). It contains Cu^2+^ in square-planar coordination, CuO_2_ fragments are only edge-shared forming ribbon-like chains. The Sr_2_CuO_3_ crystal structure is heavily oxygen deficient and, therefore, Cu is in square-planar coordination and CuO_2_ units are corner-shared. Sr_14_Cu_24_O_41_ has a composite crystal structure which consists of two incommensurate sublattices, first contains (Cu_2_O_3_) ladders and second contains Cu-O 1D edge-shared chains similar to SrCuO_2_ (Figure 5). Therefore, it contains spin-ladder fragment as in 80 K superconductor YBa_2_Cu_4_O_8_ [68,69].

Members of the spin-ladder homologous series Sr_n−1_Cu_n+1_O_2n_ (*n* = 3, 5), Sr_2_Cu_4_O_6_ and Sr_4_Cu_6_O_10_, are stabilized by high-pressure because they have elevated crystallographic density in comparison with normal pressure phase Sr_14_Cu_24_O_41_ (5.51 g/cm^3^, 5.61 g/cm^3^ and 4.68 g/cm^3^), respectively.

**Figure 6:**
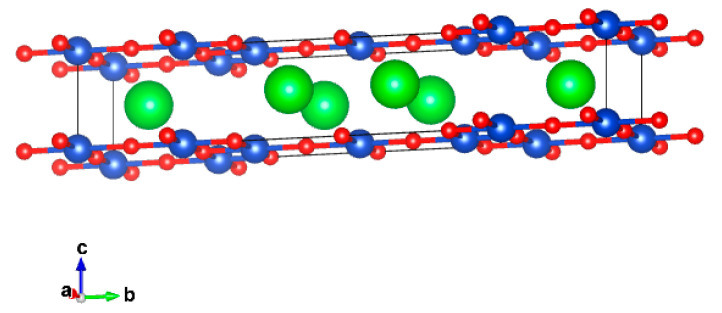
Crystal structure of Sr_4_Cu_6_O_10_. Sr, Cu and O atoms are shown by green, blue and red circles, respectively.

These first and second series members with *n* = 3 and *n* = 5 were synthesized in bulk form at 6 GPa and 1200 °C, the next member with *n* = 7 was not obtained yet, although it seems that it could be obtained at higher pressures [71]. Refinement of Sr_4_Cu_6_O_10_ crystal structure using neutron powder diffraction data confirmed the 3-leg model (see Figure 6). The Cu-O planes consist of square planar CuO_2_ units which are linked by both edge- and corner-sharing, corner-sharing chains of CuO_2_ squares form the legs of the ladder, running in the *a*-direction. These ladders are connected by edge-sharing forming double chains.

Single crystals of Sr_4_Cu_6_O_10_ slightly doped by Ca (*ca.* 4%) were grown using a high argon-gas-pressure technique with an Ar partial gas pressure of 1.82 GPa in a three-zone Canthal furnace [72]. Crystal structure determination using single crystal X-ray diffraction data confirmed the 3-leg model.

Cuprates with ladder structures are of particular interest as so-called spin-ladder compounds. There are theoretical predictions of superconductivity or charge- density-wave properties for properly doped spin-ladder materials with an even number of legs [73,74]. However, up to now, bulk superconductivity was never observed in spin-ladder cuprates. All attempts to induce superconductivity by appropriate electron and hole-doping (by La^3+^, Nd^3+^, Na^+^ and K^+^) in Sr_2_Cu_4_O_6_ and Sr_4_Cu_6_O_10_ failed to give any positive results. Superconductivity in Ca-doped Sr_14_Cu_24_O_41_ was reported, but never confirmed, at least in the bulk state [75]. AFM transition was observed in the non-doped phase below 60 K and it was attributed to the spin orientation predominantly perpendicular to the 1D CuO_2_ chains [76]. Therefore, it is still an open question whether is possible to induce superconductivity by the appropriate doping in the parent spin-ladder compounds containing edge-shared CuO_2_ units even if Cu has the appropriate oxidation state

There is a considerable theoretical interest in one-dimensional 1D Heisenberg spin-1/2 systems because they exhibit a number of properties that are entirely dominated by quantum-mechanical behavior and have no analogues in three-dimensional systems. In particular, it has been shown that the Heisenberg *S*51/2 chain represents an integrable system characterized by a macroscopic number of conservation laws [77].

Some 1D cuprates showed unusual behavior at low temperatures, particularly the research efforts were focused on interesting quantum magnetic phenomena in copper-oxygen chains with integer spins. It should be mentioned the spin-Peierls transition in CuGeO_3_ [78] and the Haldane-gap appearance in Heisenberg chains with integer spins [79].

Thermal conductivities of normal pressure 1D spin-chain compounds SrCuO_2_ and Sr_2_CuO_3_ were studied in detail [80,81]. Although the crystal structures of the two compounds are different in the sense that the former contains linear and the latter consists of zigzag Cu-O chains, the thermal conductivity of both materials is remarkably similar. In particular, the heat transport in the directions perpendicular to the chains is dominated by phonons, but along the chain direction and at high temperatures there is a substantial excess contribution related to the transport of energy by spinons. The phonon thermal conductivity is analyzed employing a Debye-type approximation. The main sources of phonon scattering are phonons at high temperatures and lattice defects, presumably dislocations, at low temperatures. The spin-phonon interaction is not seen in the phonon heat transport, most likely because it is masked by other scattering processes.

**Figure 7:**
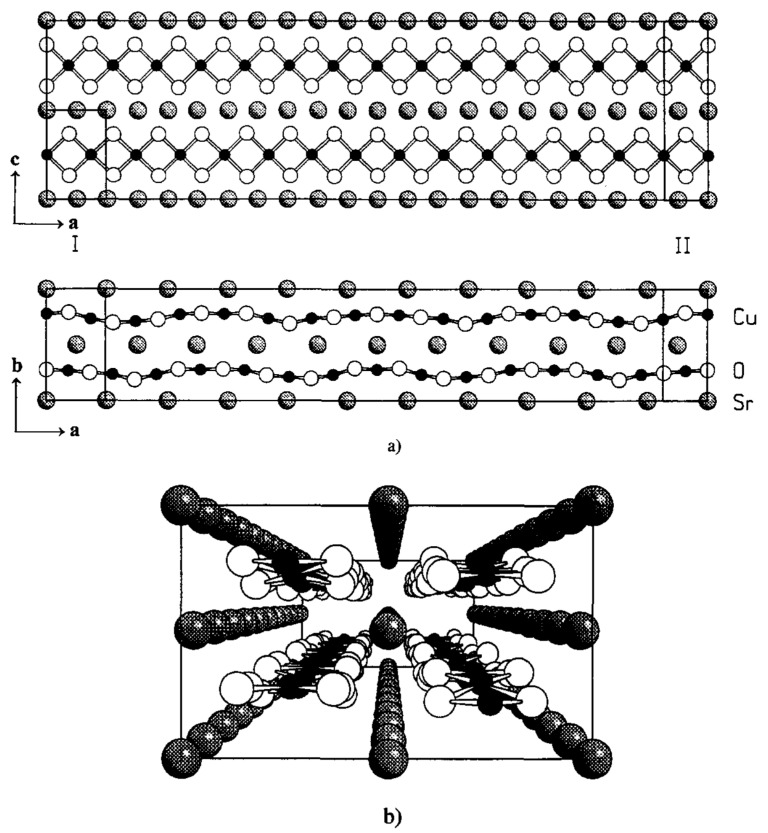
(**a**) Structure of Sr0.73CuO2 consisting of one-dimensional edge-sharing CuO2 chains alternating with Sr planes. CuO2 chains are wave-modulated. I: subcell of Sr atoms. II: subcell of Cu and O atoms. (**b**) Perspective view of the Sr0.73CuO2 structure in a direction.

As was noticed, high isostatic pressure in belt-type apparatus and cubic anvils leads to stabilization of phases with elevated crystallographic densities, but in the case of high partial oxygen pressure of 0.2 GPa in a double chamber gas system, Sr_0.73_CuO_2_ was obtained at the temperature range 950–1180 °C, single phase polycrystalline samples and single crystals were obtained [82]. Its cation and oxygen stoichiometry was confirmed by energy dispersive X-ray (EDX) analysis and volumetric determination method [83]. Its incommensurate composite crystal structure belongs to the NaCuO_2_ structural type and consists of two sublattices, associated with Sr^2+^ cations and (CuO_2_)^n−^ edge-shared square-planar units, respectively. First sublattice has an orthorhombic C-centered subcell with a = 3.78 Å, b = 6.82 Å, c = 5.51 Å and second has an orthorhombic F-centered subcell with a = 2.72 Å, b = 6.82 Å, c = 11.02 Å. Therefore, there is a mismatch between them resulting in incommensurate modulations along the *a*-axis (Figure 7).

Ca_0.83_CuO_2_ has similar composite incommensurate structure. It was obtained as nearly single-phase polycrystalline sample from precursors of Ca_2_CuO_3_ and CuO heated at 1020 °C for 36 h and oxygen partial pressure of 0.174 GPa. The oxygen stoichiometry of Ca_0.83_CuO_2_ was confirmed by volumetric titration. Single crystals were grown by using a BaCuO_2_ + CuO flux [84]. It was shown that Ca-containing phase has an incommensurate composite crystal structure similar to Sr_0.73_CuO_2_. However, some peculiarities were manifested. It contains Cu in oxidation state +2.34 that is well below +2.54 revealed for Sr-containing phase. Recently, it was reported Ca_0.83_CuO_2_ synthesis at ambient pressure using very effective soft chemistry sol-gel technique in flowing oxygen at 700 °C [85].

Moreover, it was found by electron diffraction that the two sublattices, attributed to (CuO_2_)^n−^ and Ca^2+^, have the following unit cell parameters:

(1) monoclinic subcell with a = 3.33 Å, b = 6.32 Å, c = 5.47 Å, ß = 105.0°
(2) monoclinic subcell with a = 2.80 Å, b = 6.32 Å, c = 5.47 Å, ß = 104.9°

The authors used the superspace approach [86]. The whole ED pattern was indexed by adopting the diffraction vector **H** = ha* + kb* + lc* + m**q**; **q** is a modulation vector. The modulation vectors were found to be 1.20144(7) a* − 0.0038(6) c* and 0.83057(7) a* + 0.0032(3) c*, for Ca and CuO_2_ subcell, respectively.

The powder diffraction data were collected using synchrotron radiation with λ = 0.685 Å. Then the crystal structure was refined using the Jana 2006 software package adopted for composite structure refinement [87].

It is important to note that the CuO_4_ square-planar units were significantly distorted due to propagation of a tilted wave along the ribbons. Cu-O distances are between 1.85 Å and 2.05 Å with an average value 1.93 Å. Taking into account the mixed Cu oxidation state, the possibility of periodic charge-ordering phenomena along the Cu-O chains is envisaged.

**Figure 8:**
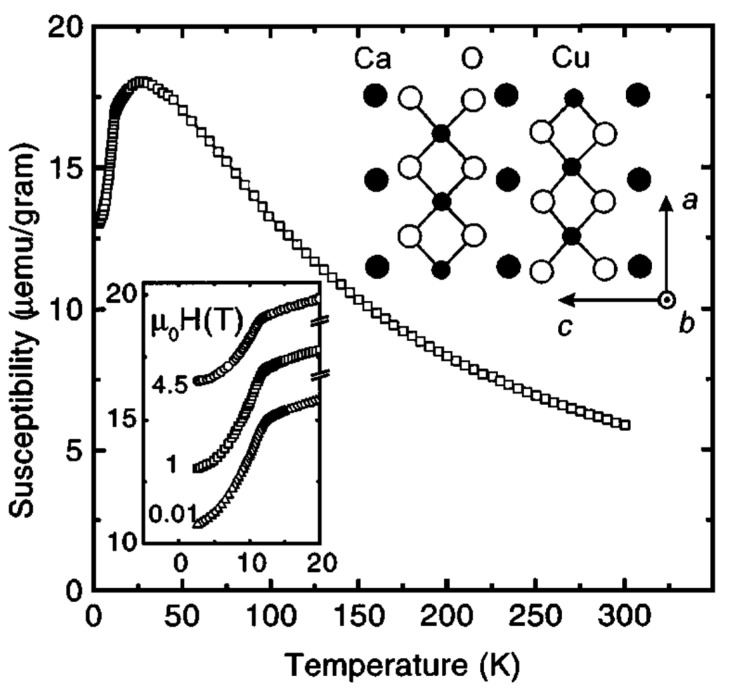
Susceptibility χ(T) of as-prepared polycrystalline Ca0.83CuO2 at µ0H = 1 T. The χ (T) curves at µ0H = 10 mT and 4.5 T are offset for clarity. The value of χ(T > 15 K) is independent from the applied field. Part of the unit cell of Ca0.83CuO2 is displayed. Reproduced with permission from [84], Figure 1.

**Figure 9:**
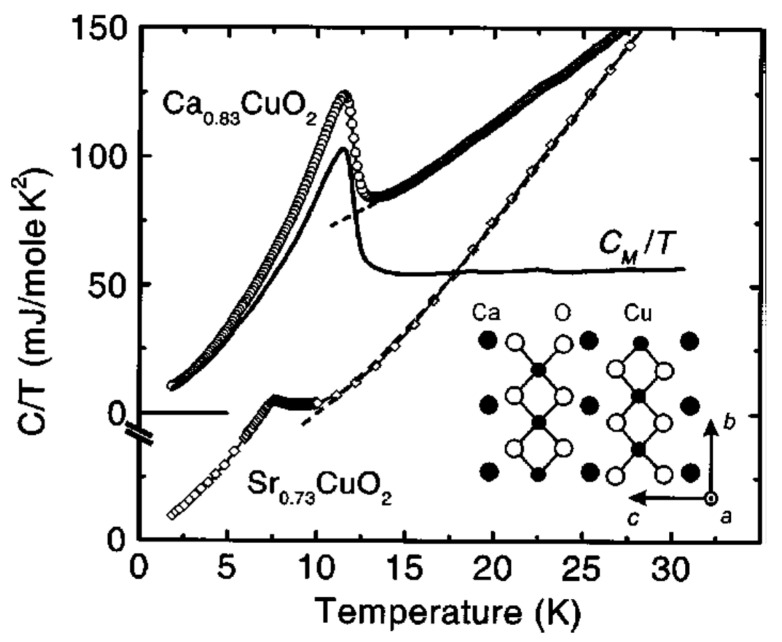
Specific heat *C*(*T*) of Ca_0.83_CuO_2_ and Sr_0.73_CuO_2_. The curves for Ca_0.83_CuO_2_ are offset for clarity. The magnetic contribution *C_M_* /*T* is shown for Ca_0.83_CuO_2_. The dashed lines are fits of (*C_M_*/*C_L_*)/*T* to the high-*T* data. Reproduced with permission from [89], Figure 1.

Magnetic properties of Sr_0.73_CuO_2_ and Ca_0.83_CuO_2_ were studied in details by DC magnetic susceptabilities, EPR, specific heat and elastic neutron scattering on polycrystalline samples, and magnetic torque measurements on single crystals [84,88,89]. It was reported that in Sr_0.73_CuO_2_ and Ca_0.83_CuO_2_, in contrast to Sr_14_Cu_24_O_41_, long-range 3D AF magnetic order occurs below *T_N_* ~ 12 K and ~ 10 K, respectively (Figure 8 and Figure 9). At higher temperatures, Sr_0.73_CuO_2_, Ca_0.83_CuO_2_ and Sr_14_Cu_24_O_41_ showed similar magnetic properties: CuO_2_ chains in both compounds are diluted highly magnetically by holes which render the CuO_2_ unit nonmagnetic.

A Ba-containing composite phase was reported as well, its formula was expressed as Ba_2_Cu_3_O_5+d_ and the crystal structure was briefly described [90]. It has an incommensurable modulated structure probably similar to Sr_0.73_CuO_2_ and Ca_0.83_CuO_2_, but the structure details and physical properties still need to be carefully investigated.

## 6. Importance of High-Pressure for Synthesis of New Oxide Superconductors and Future Prospects

It is particularly important to underline the crucial role of high pressure in the synthesis of layered cuprates. It is briefly summarized in Table 1.

In general, solid-state synthesis in most cases can only be performed at high temperatures. That may be a negative factor, in two cases: preparation of low-temperature structural forms and stabilization of metastable materials [91,92]. These problems can be solved by using high pressure, especially if the final product has a smaller volume, negative value of ΔV (and, therefore, higher crystallographic density) that the precursors mixture. Indeed, CaCuO_2_ with “infinite-layer” structure has slightly higher density than the mixture of precursors Ca_2_CuO_3_ + CuO, the difference is about 10%, that’s why this metastable compound is strongly stabilized by high-pressure. Another example is the low-temperature superconductor (T_c_ = 2 K) KOs_2_O_6_ with partially A-deficient cubic pyrochlore-like structure that at 3 GPa and 900 °C transforms into a triclinic structure with higher density [93,94].

**Figure 10:**
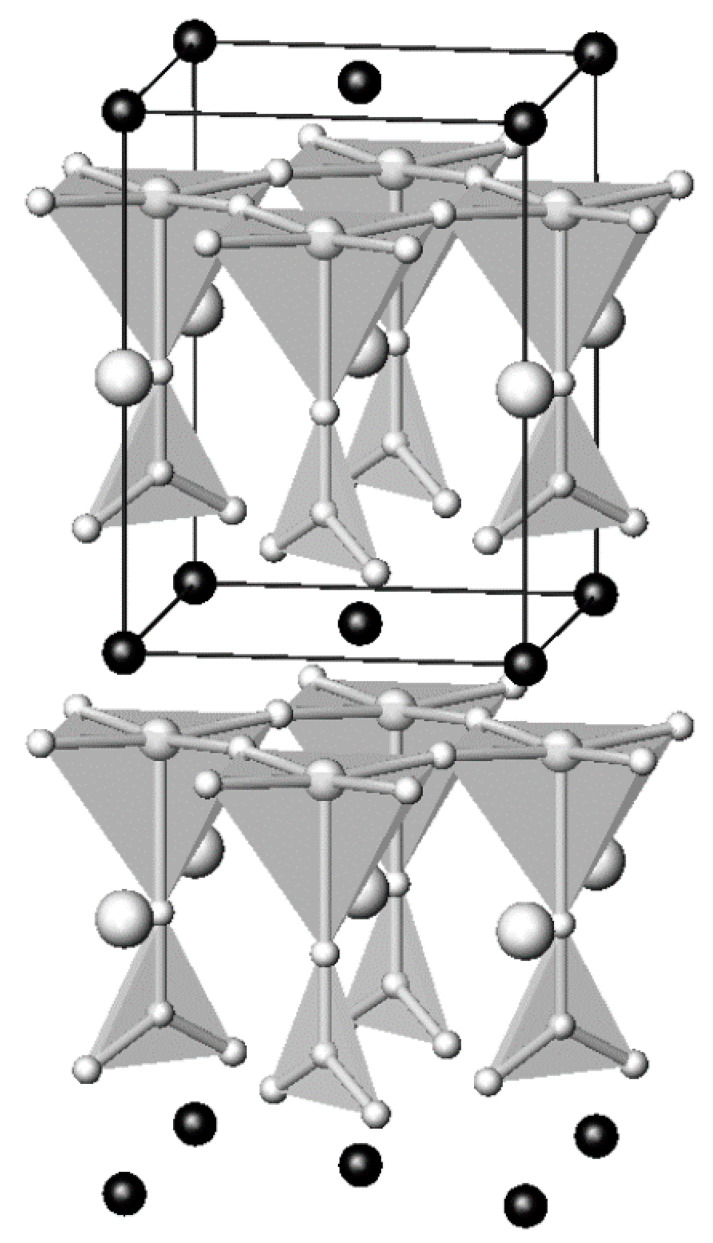
Crystal structure of NdBaCuO_2_BO_3_. Cu (pyramid) and B (triangular) coordination polyhedral are emphasized. Ba and Nd atoms are shown by light and dark circles, respectively. Reproduced with permission from [99], Figure 4.

It is well known that high-pressure application increases the chemical reactivity of precursors and accelerates the kinetics of solid-state reactions. This is particularly important for the synthesis of higher members of homologous series, because usually the kinetics of these reactions are very slow and occur via formation of lower series members as intermediary products. Moreover, high pressure suppresses the decomposition of thermally unstable precursors. These three important factors, negative ΔV value, increase of precursors thermal stability and acceleration of kinetics of solid-state reactions open large space for synthesis of novel materials. The example can be stabilization of C-containing layered cuprates and synthesis of corresponding homologous series C(Sr_2−x_Ca_x_)_2_(Ca,Ln)_n−1_Cu_n_O_2n+2_ (*n* = 1–4; Ln = rare-earth cations) and similar B-containing series at high pressures. Their crystal structures contain triangular oxycarbonate (CO_3_)^2−^ and oxyborate (BO_3_)^3−^ fragments alternate with perovskite-like blocks along the *c*-axis [95,96,97,98,99,100,101,102]. The first members, CSr_2_CuO_5_ (but not CCa_2_CuO_5_) and RBaCuO_2_BO_3_ (R = La-Eu), were obtained at ambient pressure. They have weak tetragonal superstructure with *a_s_ = a√2, c_s_ = 2a*, due to an ordering of triangular oxycarbonate (CO_3_)^2-^ and oxyborate (BO_3_)^3−^ structural fragments with alternating orientation and for that reason it is not detectable from X-ray diffraction data. Structural peculiarities of NdBaCuO_2_BO_3_ were revealed from neutron powder diffraction data (Figure 10). It should be noted that the boron atom has an almost ideal triangle coordination with a B-O average distance of ~1.38 Å and it is considerably longer that corresponding C-O average distance equal to ~1.27 Å [95].

C and B-contained 1201 crystal structures are similar, but the space group of NdBaCuO_2_BO_3_ is non-centrosymmetric P4bm due to an ordering of Nd^3+^ and Ba^2+^ cations that was deduced from TEM data.

Moreover, a (Cu,N,C)Sr_2_Ca_n−1_Cu_n_O_y_ series with more complicated composition was reported. Its four members (*n* = 3–6) were synthesized in a belt-type apparatus at 6 GPa and 1350 °C for 1–6 h [103]. It is interesting that only some discreet members (*n* = 2, 5) were obtained in nitrogen-free systems, and even the small amount of N-doping strongly stabilizes the series.

Incorporation of nitrate to the crystal structures of (Cu,N,C)Sr_2_Ca_n−1_Cu_n_O_y_ was confirmed by EELS [104]. It is very interesting that the two different superstructures were observed simultaneously in the case of *n* = 3, *2a × b × 2c* and 4*a × b × 2c.* For the next member (*n* = 4) only the first type of superstructure was revealed and no evidence of superstructure was found for the members with *n* = 5 and 6.

The two first members of this series were synthesized at slightly lower pressure and temperature, 5.5 GPa and 1270 °C [105]. Contrary to non-superconducting CSr_2_CuO_5_ and its Ca-substituted analog, the sample with nominal initial composition C_0.8_N_0.2_Sr_2_CuO_5.3_ exhibited bulk superconductivity with T_c_ = 33 K, however the doping mechanism is still remains unclear. If we consider the presence of pentavalent nitrogen in nitrate (NO_3_)^−^ form, it is logical to expect electron doping. However, two known families of cuprate superconductors with electron as a hole carrier are SrCuO_2_ with infinite-layer structure doped by rare-earth trivalent cation (La-Eu) and R_2_CuO_4_ (R = Nd-Gd) doped by Ce^4+^, so-called T’-phase [106,107,108]. Typical Cu-O in-plane distance is 1.97–1.98 Å. Both contain Cu in a square-planar coordination. In CSr_2_CuO_5_ crystal structure copper is in square-pyramidal coordination, Cu-O in-plane distance is much shorter and below 1.95 Å, so the scenario of electron-doped superconductivity seems very improbable in this case.

All reported members of the (Cu,N,C)Sr_2_Ca_n−1_Cu_n_O_y_ series exhibited superconducting transitions with T_c_ = 33, 91, 90, 113, 65 and 52 K, for the members with n = 1, 2, 3, 4, 5 and 6, respectively. It should be pointed out that (Cu,N,C)-1212-Sr phase has a typical *4a × b × 2c* superstructure the same as (Cu,C)-1212-Sr originating from Cu-C-C-C-type ordering in the (Cu,C)-plane. However, X-ray single crystal studies of CCa_2_CuO_5_ and CSr_1.9_Ca_1.1_Cu_2_O_7_ did not reveal any additional reflexes corresponding to the above- mentioned superstructures [109]. It would be interesting to grow and study N-doped single crystals and to perform a neutron powder diffraction study to investigate in detail of light atoms coordination and interatomic distances.

It can conclude that the application of high-pressure, indeed, has a large potential for the realization of different compounds of various structure types, some of which can be only stabilized under high-pressure conditions.

However, the principal question is to how define an efficient working tool that will provide a reliable prediction of new compounds with high T_c_. Combinatorial solid-state chemistry is proved to be an efficient way to search for new superconducting compounds, but the related problem of identification of diamagnetic phases in polyphasic samples is not easily solved. In [110], samples were synthesized by solid state reactions in a system of randomly mixed starting components (Ca, Sr, Ba, La, Y, Pb, Bi, Tl, and Cu oxides). They showed an onset of diamagnetic transition above 115 K in bulk measurements. Imaging of this diamagnetic response in ceramic samples by scanning SQUID microscopy (SSM) revealed local superconducting areas with sizes down to as small as the spatial resolution of a few micrometers. In addition, locally formed superconducting phases were extracted from polyphasic samples by magnetic separation. The analysis of single grains (d < 80 mm) by X-ray diffraction, elemental analysis, and SQUID measurements allowed to identity Tl_2_Ca_3_Ba_2_Cu_4_O_12_, TlCaBaSrCu_2_O_7−δ_, BaPb_0.5_Bi_0.25_Tl_0.25_O_3−δ_, TlBa_2_Ca_2_Cu_3_O_9_, Tl_2_Ba_2_CaCu_2_O_8_, and YBa_2_Cu_3_O_7_ well-known superconducting phases with high T_c_.

In another publication, the authors introduced two different approaches such as the high-pressure, high-temperature method and ceramic combinatorial chemistry and reported their application to several typical examples [111]. The authors demonstrated that a single sample synthesis concept based on multielement ceramic mixtures can produce a variety of local products. This concept should include local probe analyses and separation techniques to identify compounds of interest. The authors presented the results obtained by applying the new concept to random mixtures of Ca, Sr, Ba, La, Zr, Pb, Tl, Y, Bi, and Cu oxides reacted at different conditions. By adding Zr but removing Tl, Y, and Bi, the bulk state superconductivity got enhanced up to about 122 K.

Another very promising way includes a methodology for crystal structure prediction that is based on the evolutionary algorithm USPEX and the machine-learning interatomic potentials actively learning on-the-fly [112]. It allows for an automated construction of an interatomic interaction model from scratch, replacing the expensive density functional theory (DFT) and giving a speedup of several orders of magnitude. This methodology was successfully tested on prediction of crystal structures of carbon, high-pressure phases of sodium, and boron allotropes, including those that have more than 100 atoms in the primitive cell.

Based on this algorithm, two high-T_c_ hydride superconductors ThH_9_ (T_c_ = 149 K) and ThH_10_ (T_c_ = 159–161 K) were predicted and synthesized at 170–175 GPa [113,114]. It is important to note that ThH_10_ with cubic *fcc* structure has very wide interval of stability from 85 to 185 GPa.

Moreover, a new non-empirical method for the prediction of material(s) among all possible combinations of all elements has been reported recently [115]. It was stated that this method possesses the best combination of target properties because it combines a new coevolutionary approach with the carefully restructured “Mendelevian” chemical space, energy filtering, and Pareto optimization to ensure that the predicted materials have optimal properties and a high chance to be synthesizable. The approach was supported by the first calculations, in particular, it was found that diamond (and its polytypes, including lonsdaleite) are the hardest possible materials and that *bcc*-Fe has the highest zero-temperature magnetization among all possible compounds. There is no doubt that these two methodologies are very promising in the search for new superconducting materials.

## 7. Conclusions

Synthesis, crystal structures and magnetic properties of several families of layered cuprates were discussed. These materials are superconductors or related phases. It is underlined that high-pressure is the most important tool and its application opens large prospects for design and synthesis of new superconducting materials, particularly when used together with combinatorial solid state chemistry and advanced methodologies for prediction of new phases with high-T_c_.

## Figures and Tables

**Table 1:** Series of cuprates containing structural flat fragments: high-pressure (HP) synthesis and superconductivity.

General Formula	HP for Synthesis	Superconductivity
I.HgBa_2_Ca_n−1_Cu_n_O_2n+2+__δ_ (*n* = 1–6)	HP is required for *n* = 4–6.	T_c_ max = 138 K (*n* = 3), up to 166 K in situ at > 20 GPa
II.AuBa_2_(Ca,Ln)_n−1_Cu_n_O_2n+3_ (*n* = 1–4, Ln = rare-earth cations)	HP is required	T_c_ max = 99 K (*n* = 4)
III.(La,M)_n+1_Cu_n_O_2n+2±__δ_ (RP- related):		
1.(La,M)_n+1_Cu_n_O_2n+2+__δ_ (M = Ca, Sr, Ba)	HP isn’t always required for *n* = 1, 2	T_c_ max = 38 K (*n* = 1), up to 52.5 K in situ at 1.68 GPa.
		T_c_ max ~ 100 K for *n* = 2 (as minor phase)
2. Sr_2-x_Ba_x_CuO_3+ô_	HP with internal oxidizer is required	T_c_ max = 98 K at x = 0.6 (bulk ?)
3. Ba_2_CuO_4−y_	18 GPa with internal oxidizer is required	T_c_ max > 70 K at y = 0.8 (bulk), possibly original mechanism
IV.(Cu,A)(Sr,M)_2_(M,Ln)_n−1_Cu_n_O_y_ (A = B, C, N; M= Ca, Sr, Ba; Ln = rare-earth cations; *n* = 1–6)	HP is required at least for *n* = 3–6 members	T_c_ max = 113 K for (Cu,N,C)Sr_2_Ca_n−1_Cu_n_O_y_ (*n* = 4)
V. Sr_n−1_Cu_n+1_O_2n_ (*n* = 3,5)	HP is required	Not superconducting
